# Recombinant human activated protein C ameliorates oleic acid-induced lung injury in awake sheep

**DOI:** 10.1186/cc7128

**Published:** 2008-11-20

**Authors:** Kristine Waerhaug, Mikhail Y Kirov, Vsevolod V Kuzkov, Vladimir N Kuklin, Lars J Bjertnaes

**Affiliations:** 1 Department of Anesthesiology, Institute of Clinical Medicine, Faculty of Medicine, University of Tromsø, 9037 Tromsø, Norway; 2 Department of Anesthesiology, Northern State Medical University, Troitzky avenue 51, 163000 Arkhangelsk, Russian Federation

## Abstract

**Introduction:**

Acute lung injury (ALI) may arise both after sepsis and non-septic inflammatory conditions and is often associated with the release of fatty acids, including oleic acid (OA). Infusion of OA has been used extensively to mimic ALI. Recent research has revealed that intravenously administered recombinant human activated protein C (rhAPC) is able to counteract ALI. Our aim was to find out whether rhAPC dampens OA-induced ALI in sheep.

**Methods:**

Twenty-two yearling sheep underwent instrumentation. After 2 days of recovery, animals were randomly assigned to one of three groups: (a) an OA+rhAPC group (n = 8) receiving OA 0.06 mL/kg infused over the course of 30 minutes in parallel with an intravenous infusion of rhAPC 24 mg/kg per hour over the course of 2 hours, (b) an OA group (n = 8) receiving OA as above, or (c) a sham-operated group (n = 6). After 2 hours, sheep were sacrificed. Hemodynamics was assessed by catheters in the pulmonary artery and the aorta, and extravascular lung water index (EVLWI) was determined with the single transpulmonary thermodilution technique. Gas exchange was evaluated at baseline and at cessation of the experiment. Data were analyzed by analysis of variance; a *P *value of less than 0.05 was regarded as statistically significant.

**Results:**

OA induced profound hypoxemia, increased right atrial and pulmonary artery pressures and EVLWI markedly, and decreased cardiac index. rhAPC counteracted the OA-induced changes in EVLWI and arterial oxygenation and reduced the OA-induced increments in right atrial and pulmonary artery pressures.

**Conclusions:**

In ovine OA-induced lung injury, rhAPC dampens the increase in pulmonary artery pressure and counteracts the development of lung edema and the derangement of arterial oxygenation.

## Introduction

Mortality from acute lung injury (ALI) still remains between 30% and 40% [1]. Patients with acute respiratory distress syndrome (ARDS), the most severe form of ALI, present with elevated plasma concentrations of oleic acid (OA), which is one of the most abundantly occurring fatty acids in human plasma [2]. The proportion of OA increases in the bronchoalveolar lavage fluid of patients with pneumonia and ARDS [3]. In combination with sepsis, an enhanced plasma level of OA adds to the risk of contracting ARDS [4]. However, independent of whether a high plasma concentration of OA contributes to ARDS or not, infusion of OA has been widely used to mimic non-septic lung injury in experimental settings. Administered to animals, OA increases pulmonary vascular pressure and permeability, resulting in the development of lung edema and arterial hypoxemia that are typical for this condition [5].

Activated protein C (APC) antagonizes thrombin generation by inactivating coagulation factors Va and VIIIa with protein S as a co-factor [6,7]. It has been suggested that, by binding to endothelial APC receptor (EPCR) and to protease activated receptor-1 (PAR-1), APC initiates cytoprotective reactions, gene expression profile alterations, and anti-inflammatory and anti-apoptotic effects [8-11].

Recombinant human APC (rhAPC) increases survival from severe sepsis [12]. Reportedly, patients receiving rhAPC demonstrate a shorter duration of respiratory failure [13]. Protein C decreases markedly in patients with ALI, whether of septic or non-septic origin, and a low plasma level of protein C is associated with a poor clinical outcome [14,15]. We speculated that rhAPC could be of potential benefit in the treatment of ALI. Recent studies of ovine sepsis or endotoxin-induced ALI support this assumption [16-18], whereas others have failed to demonstrate favorable effects either in animal [19-21] or human [22] studies. Up to now, no one study has documented beneficial effects of APC on models of non-septic ALI. The aim of the present study was to investigate whether intravenously administered rhAPC alleviates ovine OA-induced lung injury, assessing changes in pulmonary hemodynamics, extravascular lung water, and arterial oxygenation.

## Materials and methods

The Norwegian Experimental Animal Board approved the study according to the rules and regulations of the Helsinki Convention for Use and Care of Animals.

### Animal instrumentation

Twenty-two yearling sheep weighing 34.3 ± 7.5 kg (mean ± standard deviation) were instrumented under general anesthesia and treated postoperatively as described previously by our group [23]. In brief, an 8.5-Fr introducer (CC-350B; Baxter, Deerfield, IL, USA) was inserted percutaneously in the left external jugular vein and a 5-Fr introducer (CP-07511-P; Arrow International, Inc., Reading, PA, USA) was inserted into the ipsilateral common carotid artery. After 1 to 3 days of recovery, the sheep were placed in an experimental pen. A flow-directed thermal dilution catheter (131HF7; Baxter) was introduced into the pulmonary artery, and a 4-Fr thermistor catheter (PV2014L16; Pulsion Medical Systems, München, Germany) was introduced into the thoracic aorta. The catheters were connected to pressure transducers (Transpac^®^III; Abbott Laboratories, Abbott Park, North Chicago, IL, USA) and PV8115 (Pulsion Medical Systems).

### Measurements and samples

Measurements were performed at 1-hour intervals. Mean pulmonary arterial pressure (PAP), pulmonary arterial occlusion pressure (PAOP), and mean right atrial pressure (RAP) were recorded on a Gould Polygraph 6600 (Gould Instruments, Cleveland, OH, USA). The pulmonary capillary micro-occlusion pressure (Pmo) was determined as described previously [24].

Heart rate (HR), mean systemic arterial pressure (MAP), cardiac index (CI), systemic vascular resistance index (SVRI), extravascular lung water index (EVLWI), and blood temperature were determined using a PiCCO *plus *monitor (Pulsion Medical Systems), where EVLWI is calculated using the transpulmonary thermodilution technique. Every value was calculated as a mean of three measurements, each consisting of a 10-mL bolus of ice-cold saline injected into the right atrium randomly during the respiratory cycle.

Left ventricular stroke work index (LVSWI) was calculated as LVSWI = 0.0136 × (MAP - PAOP) × CI/HR, and right ventricular stroke work index (RVSWI) was calculated as RVSWI = 0.0136 × (PAP - RAP) × CI/HR. Stroke volume index and pulmonary vascular resistance index (PVRI) were calculated using standard formulas.

Blood samples were drawn from the systemic (a) and the pulmonary artery (v) lines and analyzed for blood gases and hemoglobin (Rapid 860; Chiron Diagnostics Corporation, East Walpole, MA, USA) at the beginning and the end of the 2-hour experiment. Assuming the hemoglobin oxygen binding capacity to be 1.34 mL/g, oxygen delivery index (DO_2_I), oxygen consumption index (VO_2_I), venous admixture (Qs/Qt), and the alveolar-arterial oxygen tension difference (AaPO_2_) were calculated as described previously [23].

### Experimental protocol

After 2 days of recovery, animals were randomly assigned to one of three groups: an OA+rhAPC group (n = 8) receiving OA (Sigma-Aldrich, St. Louis, MO, USA) 0.06 mL/kg infused over the course of 30 minutes in parallel with an intravenous infusion of rhAPC (Xigris^®^; Eli Lilly and Company, Indianapolis, IN, USA) 24 μg/kg per hour during the whole 2-hour experiment, an OA group (n = 8) receiving OA as above, or a group of sham-operated animals (n = 6). All sheep received a continuous infusion of isotonic saline at 5 mL/kg per hour. After completion of the experiment, the sheep were killed with an intravenous injection of thiopental sodium (Abbott) 100 mg/kg followed by 50 mmol KCl (B. Braun Melsungen AG, Melsungen, Germany).

### Statistical analysis

Data are expressed as the mean ± standard error of the mean and analyzed by two-factor analysis of variance for repeated measurements. If F was statistically significant, Scheffe's test was applied for *post hoc *analysis of the changes in time. Comparison between OA and OA+rhAPC groups was evaluated at baseline (0 hours) and after 2 hours, applying the *t *test or the Mann-Whitney test when appropriate (SPSS 15.0 for Windows; LEAD Technologies, Charlotte, NC, USA). We regarded *P *values of less than 0.05 as statistically significant.

## Results

**Figure 1:**
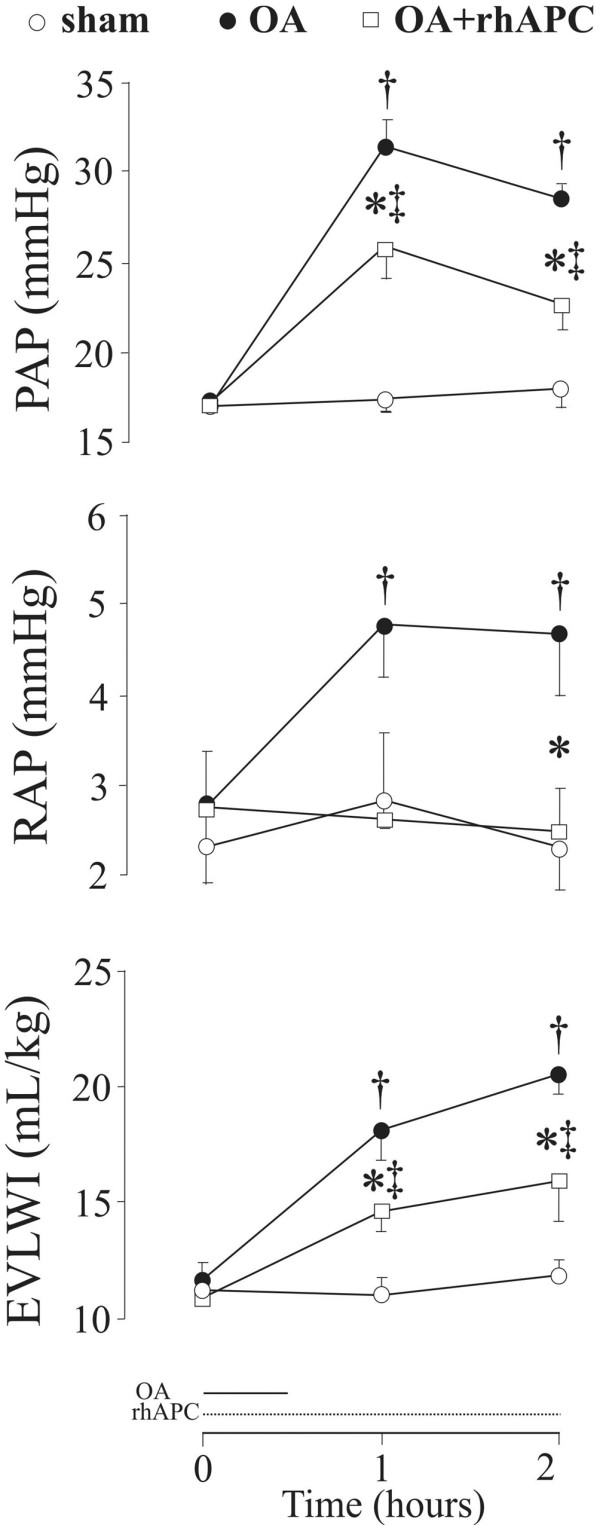
Changes in pulmonary artery pressure (PAP), right atrial pressure (RAP), and extravascular lung water index (EVLWI) in awake instrumented sheep subjected to intravenous bolus injection of oleic acid (OA) and co-administration of recombinant human activated protein C (rhAPC). In the figure, OA refers to the oleic acid-alone group (n = 8), OA+rhAPC refers to the rhAPC-treated OA group (n = 8), and sham refers to sham-operated animals (n = 6). Data are presented as mean ± standard error of the mean. *P < 0.05 between OA and OA+rhAPC groups; ^†^*P *< 0.05 from t = 0 hours in the OA group; ^‡^*P *< 0.05 from t = 0 hours in the OA+rhAPC group.

All of the sheep survived the instrumentation and the experiment without complications. Infusion of OA induced increments in EVLWI, PAP, and RAP that all declined significantly during infusion of rhAPC (Figure 1). Moreover, MAP and SVRI increased significantly (by 8% and 38%, respectively) with a concomitant 25% decrease in CI, but none of these variables was significantly influenced by rhAPC (Table 1). As the only variable, PVRI differed between the groups at baseline (*P *< 0.05). Administration of rhAPC tended to reduce the OA-induced increase in PVRI (Table 1), albeit without reaching statistical difference (*P *= 0.07). As shown in Table 1, the OA-induced changes in PAOP, Pmo, and RVSWI remained unaffected by rhAPC. We noticed no significant changes in LVSWI upon infusion of OA.

**Table 1:** Effects of recombinant human activated protein C on oleic acid-induced changes in systemic and pulmonary hemodynamics in awake sheep

	Time		
	0 hours	1 hour	2 hours

MAP, mm Hg			
Sham	99 ± 4	99 ± 5	98 ± 5
OA	94 ± 3	102 ± 2	104 ± 3^†a^
OA+rhAPC	94 ± 3	99 ± 3	98 ± 4
CI, L/min per m^2^			
Sham	5.8 ± 0.2	5.5 ± 0.2	5.3 ± 0.2
OA	6.3 ± 0.3	4.8 ± 0.4^a^	4.7 ± 0.5
OA+rhAPC	5.8 ± 0.4	4.5 ± 0.3^a^	4.2 ± 0.3^a^
SVRI, dynessecm^2^/cm^5^			
Sham	1,627 ± 58	1,497 ± 97	1,479 ± 153
OA	1,231 ± 65	1,870 ± 320	1,696 ± 130^a^
OA+rhAPC	1,326 ± 69	1,734 ± 132^a^	2,085 ± 220^a^
PAOP, mm Hg			
Sham	9 ± 0	8 ± 0	9 ± 1
OA	9 ± 0	13 ± 1^a^	13 ± 1^a^
OA+rhAPC	8 ± 0	12 ± 1^a^	11 ± 1^a^
Pmo, mm Hg			
Sham	7 ± 0	7 ± 0	7 ± 0
OA	7 ± 1	10 ± 1	12 ± 1^a^
OA+rhAPC	7 ± 1	11 ± 1^a^	10 ± 1^a^
PVRI, dynessecm^2^/cm^5^			
Sham	112 ± 12	134 ± 15	136 ± 9
OA	104 ± 8	333 ± 59^a^	300 ± 39^a^
OA+rhAPC	132 ± 8^b^	233 ± 25^a^	231 ± 28^a^
HR, beats per minute			
Sham	104 ± 6	103 ± 6	93 ± 6
OA	109 ± 5	106 ± 12	104 ± 12
OA+rhAPC	99 ± 6	95 ± 8	84 ± 5^a^
SVI, mL/beat per m^2^			
Sham	58 ± 4	55 ± 2	60 ± 6
OA	59 ± 5	48 ± 5	47 ± 6
OA+rhAPC	58 ± 5	49 ± 4^a^	50 ± 4^a^
LVSWI, gm/m^2^			
Sham	70 ± 4	67 ± 3	71 ± 2
OA	69 ± 8	57 ± 5	59 ± 8
OA+rhAPC	68 ± 3	57 ± 4	59 ± 5
RVSWI, gm/m^2^			
Sham	11 ± 1	11 ± 0	12 ± 1
OA	12 ± 1	17 ± 2^a^	15 ± 1
OA+rhAPC	11 ± 1	15 ± 1	14 ± 1

Values are presented as mean ± standard error of the mean. Sham refers to sham-operated sheep (n = 6), OA refers to sheep receiving infusion of oleic acid (n = 8), and OA+rhAPC refers to sheep receiving oleic acid and recombinant human activated protein C (n = 8). ^a^*P *< 0.05 from t = 0 hours. ^b^*P *< 0.05 between OA and the OA+rhAPC groups. CI, cardiac index; HR, heart rate; LVSWI, left ventricular stroke work index; MAP, mean arterial pressure; PAOP, pulmonary artery occlusion pressure; Pmo, pulmonary micro-occlusion pressure; PVRI, pulmonary vascular resistance index; RVSWI, right ventricular stroke work index; SVI, stroke volume index; SVRI, systemic vascular resistance index.

**Figure 2:**
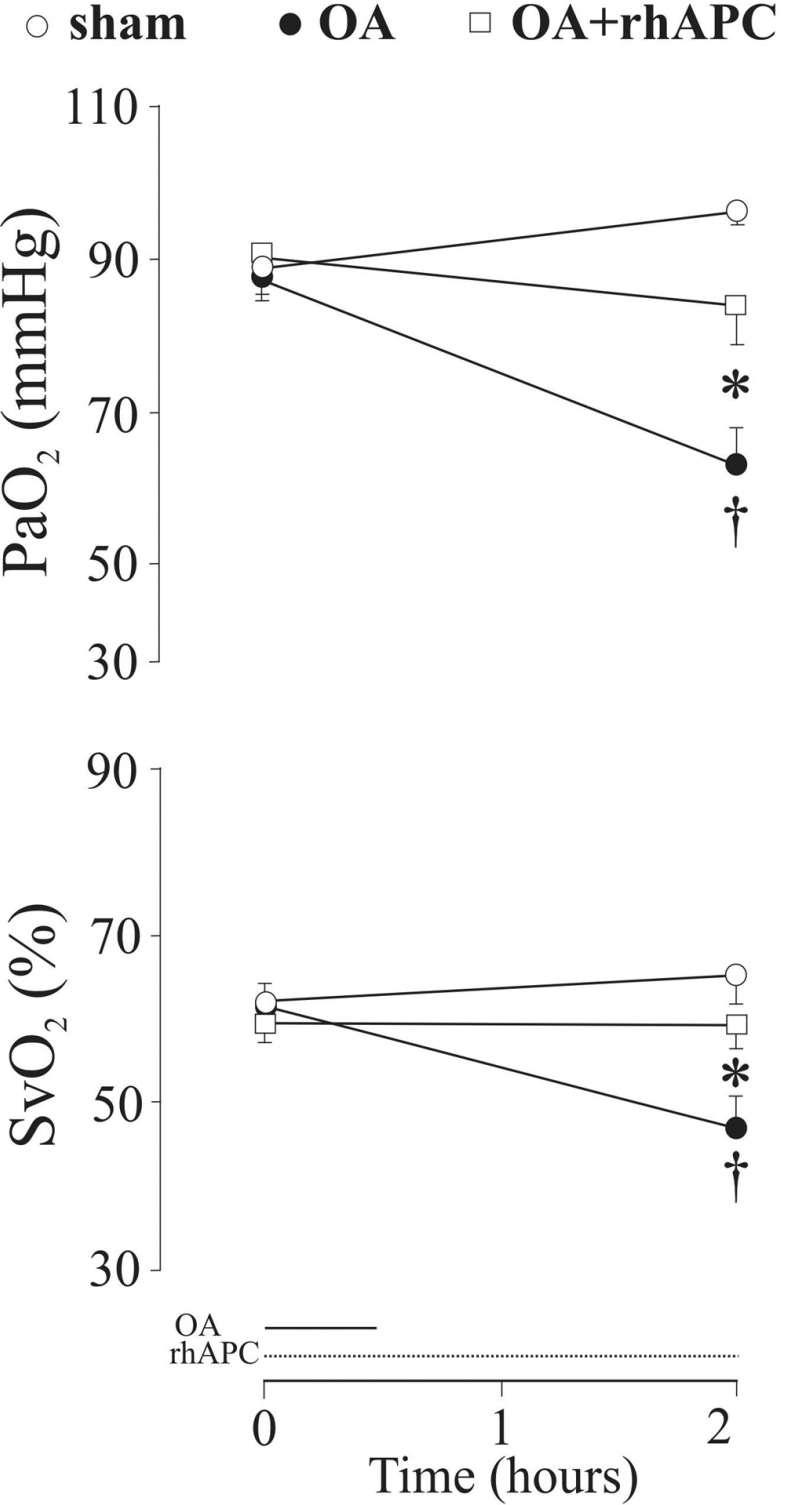
Changes in arterial oxygen partial pressure (PaO_2_) and mixed venous oxygen saturation (SvO_2_) in awake instrumented sheep subjected to intravenous bolus injection of oleic acid (OA) and co-administration of recombinant human activated protein C (rhAPC). In the figure, OA refers to the oleic acid-alone group (n = 8), OA+rhAPC refers to the rhAPC-treated OA group (n = 8), and sham refers to sham-operated animals (n = 6). Data are presented as mean ± standard error of the mean. *P < 0.05 between OA and OA+rhAPC groups; ^†^*P *< 0.05 from t = 0 hours in the OA group.

Oxygenation variables, including partial tension of oxygen in arterial blood (PaO_2_) and mixed venous oxygen saturation (SvO_2_), declined and AaPO_2 _increased after infusion of OA but improved significantly in animals exposed to rhAPC (Figure 2 and Table 2). The OA-induced increase in Qs/Qt (*P *< 0.05) (Table 2) tended to be reduced under exposure to rhAPC (*P *= 0.08) in parallel with increases in arterial oxygen saturation (SaO_2_) and pH (*P *= 0.08 and 0.06, respectively), albeit without reaching significant intergroup differences. We noticed no effect of OA on partial tension of carbon dioxide in arterial blood (PaCO_2_), blood temperature, and hemoglobin concentration, and rhAPC did not influence any of these variables (Table 2). In both groups exposed to OA, we noticed a decline in DO_2 _in comparison with baseline whereas an intragroup decrease in VO_2 _was observed only in the rhAPC-treated animals, but with no significant intergroup difference (Table 2).

**Table 2:** Effects of recombinant human activated protein C on oleic acid-induced changes in oxygen-related variables and body temperature in awake sheep

	Time	
	0 hours	2 hours

SaO_2_, percentage		
Sham	99 ± 0	99 ± 0
OA	98 ± 1	88 ± 5^a^
OA+rhAPC	99 ± 1	97 ± 1
AaPO_2_, mm Hg		
Sham	29 ± 8	27 ± 7
OA	33 ± 8	54 ± 10^a^
OA+rhAPC	28 ± 13	34 ± 12^b^
DO_2_, mL/min per m^2^		
Sham	818 ± 78	705 ± 53
OA	825 ± 39	600 ± 56^a^
OA+rhAPC	805 ± 71	566 ± 31^a^
VO_2_, mL/min per m^2^		
Sham	334 ± 38	258 ± 41
OA	316 ± 34	278 ± 110
OA+rhAPC	316 ± 75	214 ± 51^a^
O_2_ER, percentage		
Sham	38 ± 5	34 ± 3
OA	38 ± 2	47 ± 4
OA+rhAPC	40 ± 1	39 ± 2
PaCO_2_, kPa		
Sham	37 ± 2	35 ± 2
OA	36 ± 1	41 ± 4
OA+rhAPC	35 ± 1	40 ± 2
Qs/Qt		
Sham	0.042 ± 0.009	0.062 ± 0.018
OA	0.062 ± 0.014	0.246 ± 0.080^a^
OA+rhAPC	0.045 ± 0.016	0.087 ± 0.027
Arterial pH		
Sham	7.51 ± 0.02	7.52 ± 0.01
OA	7.50 ± 0.01	7.44 ± 0.02
OA+rhAPC	7.51 ± 0.01	7.50 ± 0.02
Hemoglobin, g/dL		
Sham	10 ± 1	10 ± 0
OA	10 ± 0	11 ± 0
OA+rhAPC	10 ± 0	10 ± 0
Body temperature, °C		
Sham	40.0 ± 0.2	39.5 ± 0.4
OA	39.3 ± 0.3	39.3 ± 0.3
OA+rhAPC	39.4 ± 0.1	39.3 ± 0.1

Values are presented as mean ± standard error of the mean. Sham refers to the sham-operated sheep (n = 6), OA refers to sheep receiving infusion of oleic acid (n = 8), and OA+rhAPC refers to sheep receiving oleic acid and recombinant human activated protein C (n = 8). ^a^*P *< 0.05 from t = 0 hours. ^b^*P *< 0.05 between OA and the OA+rhAPC groups. AaPO_2_, alveolar-arterial oxygen tension difference; DO_2_, oxygen delivery index; O_2_ER, oxygen extraction ratio; PaCO_2 _arterial partial pressure of carbon dioxide; Qs/Qt, venous admixture; SaO_2_, arterial oxygen saturation; VO_2_, oxygen consumption index.

## Discussion

The present investigation has shown that simultaneous administration of rhAPC ameliorates OA-induced lung injury. The rise in pulmonary artery pressure, the evolvement of lung edema, and the derangement of arterial oxygenation subsequent to intravenous bolus infusion of OA all improved significantly during co-administration of rhAPC in our ovine model of ALI.

The lung injury that we observed after infusion of OA had the same characteristics as noticed in several previous studies of this agent on larger animals, including sheep [5]. The cardiovascular instability (including the decrease in CI and the increments in pulmonary vascular pressure and RAP), the evolvement of pulmonary edema, and the reduction of arterial and mixed venous oxygenation subsequent to administration of OA are consistent with previous reports of this type of lung injury [25]. In animals exposed to rhAPC as co-treatment, the OA-induced increments in PAP and RAP decreased and PaO_2 _increased significantly. Similar observations have been made under exposure to rhAPC in other models of lung injury in sheep [16-18]. The nearly 30% decrease in oxygen delivery was caused mainly by a combined decline in SaO_2 _and CI with OA alone and almost solely by a decrease in CI in the OA+rhAPC group (Tables 1 and 2).

Our findings agree with recently reported effects of rhAPC in other ovine models of ALI [16-18]. In these studies, the animals had been exposed to combined smoke inhalation and airway instillation of live bacteria [16], feces into the peritoneum [17], or intravenously infused endotoxin [18]. All three of the investigations demonstrated improved arterial oxygenation and dampened pulmonary hypertension in animals treated with rhAPC. However, only sheep subjected to peritoneal sepsis or endotoxin infusion presented with reduced extravascular lung water [17,18]. In contrast, Richard and colleagues [20], studying OA-induced lung injury in anesthetized mechanically ventilated pigs, found no beneficial effects of rhAPC given as pretreatment. In that study, pulmonary hemodynamics and arterial oxygenation deteriorated and plasma concentrations of IL-6 and IL-8 increased in animals subjected to infusion of rhAPC. However, our sheep had more pronounced hypoxemia as compared with their pig model. In addition, we suspect that the timing of APC pretreatment might have played a role in the outcome of the study. Possibly, the anticoagulant effects of APC could be a disadvantage before the onset of ALI. This suggestion is supported by investigators who found increased lung edema formation in rats subjected to intratracheal instillation of live *Pseudomonas aeruginosa *and co-administration of rhAPC [21]. These authors speculate that initial fibrin deposition might have sealed off the lung vasculature of non-treated animals, thereby reducing endothelial leakage.

Determination of EVLWI by means of the transpulmonary thermodilution technique is still debated. Our group and others have compared transpulmonary thermodilution with both the thermo-dye dilution technique and postmortem gravimetry and demonstrated close correlations [26-28].

The mechanism by which APC improves OA-induced lung injury is puzzling. Experimental studies have demonstrated that neutrophils rapidly enter the pulmonary parenchyma after initiation of ALI via different mechanisms, such as hypovolemic shock [29], intestinal ischemia/reperfusion [30], or administration of endotoxin [29,31]. Thus, pulmonary neutrophil infiltration seems to be an important contributor to lung inflammation of various etiologies [32]. Inhibition of neutrophil chemotaxis and monocyte production of pro-inflammatory cytokines have been proposed to contribute to the beneficial effects of APC in sepsis and ALI [18,33-36]. However, in OA-induced ALI, neutrophil depletion does not seem to significantly affect the course of injury [37]. Early investigators noticed that OA triggers permeability edema in isolated dog lungs to which the perfusate had been depleted of blood components [38]. Therefore, most likely, the protective effects of APC on OA-induced lung injury result from intervention on other inflammatory pathways. It has been demonstrated that OA activates both the endothelin [39] and the eicosanoid pathways, including increased secretory phospholipase A_2 _[40] and thromboxane A_2 _[41-44]. Moreover, *in vitro *studies have revealed that APC causes a dose-dependent inhibition of interferon-induced expression of phospholipase A_2 _[45] and upregulation of cyclooxygenase II expression in endothelial cells [46]. OA may also promote ALI by increasing the ratio between angiotensin-converting enzymes I and II [47], upregulating inducible nitric-oxide synthase (iNOS), and inhibiting alveolar epithelial Na,K-ATPase activity [48]. In rats subjected to cecal ligation and puncture, the investigators found that depletion of protein C was associated with lung injury, upregulation of iNOS, and angiotensin-converting enzyme I/II ratio, all changes that were antagonized by administration of APC [49].

When the results of this study are evaluated, some limitations must be taken into account. First, hemodynamic and volumetric monitoring in animals subjected to respiratory distress is particularly challenging awake and may have contributed to the relatively large variations we noticed in some of the parameters. Second, in other ovine studies of sepsis or endotoxemia [16-18], effects appeared 4 to 6 hours after starting the infusion of rhAPC, so we cannot exclude the possibility that the observation time was too short for some variables to display significant intergroup differences. The reason for not prolonging the experiments beyond 2 hours was that most variables changed maximally within 1 hour and then declined to reach baseline after 3 to 4 hours. Third, there is a possibility that the study was too underpowered to show differences in all variables as early as at 2 hours. Thus, Qs/Qt, SaO_2_, oxygen extraction ratio (O_2_ER), pH, HR, Pmo, and PVRI all tended to improve in sheep receiving rhAPC (*P *= 0.06 to 0.08) alone, although without reaching statistical significance. As far as PVRI is concerned, lack of effect of rhAPC eventually could be caused by a significantly higher value at baseline compared with OA alone (Table 1). When we designed the study, we had no information about effects of rhAPC on this particular lung injury model which could be used in a power analysis of sample sizes. However, by using the present data, a retrospective analysis revealed that all of the latter variables could be expected to change significantly at a power of 80% with 10 animals in each group, but animal welfare and ethical reasons motivated us to keep the experimental groups as small as possible.

## Conclusion

The present study demonstrates that rhAPC administered as co-treatment ameliorates ovine OA-induced lung injury by reducing pulmonary edema and improving oxygenation and pulmonary hemodynamics. However, further studies are warranted to elucidate the mechanisms by which APC counteracts the OA-induced lung injury.

## Key messages

• In ovine oleic acid-induced lung injury, recombinant human activated protein C (rhAPC) ameliorates the increments in pulmonary artery pressure and right atrial pressure.

• rhAPC antagonizes the enhanced extravascular lung water and the derangement in arterial oxygenation.

## Abbreviations

AaPO_2_: alveolar-arterial oxygen tension difference; ALI: acute lung injury; APC: activated protein C; ARDS: acute respiratory distress syndrome; CI: cardiac index; EVLWI: extravascular lung water index; HR: heart rate; IL: interleukin; iNOS: inducible nitric-oxide synthase; LVSWI: left ventricular stroke work index; MAP: mean systemic arterial pressure; OA: oleic acid; PaO_2_: partial tension of oxygen in arterial blood; PAOP: pulmonary arterial occlusion pressure; PAP: pulmonary arterial pressure; Pmo: pulmonary capillary micro-occlusion pressure; PVRI: pulmonary vascular resistance index; Qs/Qt: venous admixture; RAP: right atrial pressure; rhAPC: recombinant human activated protein C; RVSWI: right ventricular stroke work index; SaO_2_: arterial oxygen saturation; SVRI: systemic vascular resistance index.

## Competing interests

This study was supported by Helse Nord (project number 4001.721.477), the departments of anesthesiology of University Hospital of North Norway and the Institute of Clinical Medicine of the University of Tromsø (Tromsø, Norway), and in part by Eli Lilly and Company (Indianapolis, IN, USA). The support from Eli Lilly and Company was limited to free use of rhAPC (Xigris^®^) in the study. MYK is a member of the Advisory Board of Pulsion Medical Systems (München, Germany). The other authors declare that they have no competing interests.

## Authors' contributions

KW participated in the experiments, analyzed the data, and drafted the manuscript. MYK, VVK, and VNK participated in the design of the study and in the experiments. LJB participated in the administration and design of the study and drafted the manuscript. All authors have read and approved the final manuscript.
